# Characterization of zinc finger protein 536, a neuroendocrine regulator, using pan-cancer analysis

**DOI:** 10.1186/s40001-024-01792-w

**Published:** 2024-05-08

**Authors:** Longjin Zeng, Longyao Zhang, Chenrui Yin, Xu Chen, Xiewan Chen, Lingyou Sun, Jianguo Sun

**Affiliations:** 1 Department of Basic Medicine, Army Medical University, Chongqing, 400038 People’s Republic of China; 2 Cancer Institute, Xinqiao Hospital, Army Medical University, Chongqing, 400037 People’s Republic of China; 3 Department of Medical Affairs, Xinqiao Hospital, Army Medical University, Chongqing, 400037 People’s Republic of China

**Keywords:** ZNF536, 19q12 amplification, Mutation, DNA methylation, RNA expression

## Abstract

**Background:**

Previous studies suggested that zinc finger protein 536 (ZNF536) was abundant in the central brain and regulated neuronal differentiation. However, the role of ZNF536 in cancer has remained unclear.

**Methods:**

ZNF536 mutation, copy number alteration, DNA methylation, and RNA expression were explored using public portals. Data from The Cancer Genome Atlas (TCGA) were utilized to analyze pathways and tumor microenvironment (TME), with a focus on prognosis in both TCGA and immunotherapy pan-cancer cohorts. Methylated ZNF536 from small cell lung cancer (SCLC) cell lines were utilized to train with probes for conducting enrichment analysis. Single-cell RNA profile demonstrated the sublocalization and co-expression of ZNF536, and validated its targets by qPCR.

**Results:**

Genetic alterations in ZNF536 were found to be high-frequency and a single sample could harbor different variations. ZNF536 at chromosome 19q12 exerted a bypass effect on CCNE1, supported by CRISPR data. For lung cancer, ZNF536 mutation was associated with longer survival in primary lung adenocarcinoma (LUAD), but its prognosis was poor in metastatic LUAD and SCLC. Importantly, ZNF536 mutation and amplification had opposite prognoses in Stand Up To Cancer-Mark Foundation (SU2C-MARK) LUAD cohort. ZNF536 mutation altered the patterns of genomic alterations in tumors, and had distinct impacts on the signaling pathways and TME compared to ZNF536 amplification. Additionally, ZNF536 expression was predominantly in endocrine tumors and brain tissues. High-dimensional analysis supported this finding and further revealed regulators of ZNF536. Considering that the methylation of ZNF536 was involved in the synaptic pathway associated with neuroendocrine neoplasms, demonstrating both diagnostic and prognostic value. Moreover, we experimentally verified ZNF536 upregulated neuroendocrine markers.

**Conclusions:**

Our results showed that ZNF536 alterations in cancer, including variations in copy number, mutation, and methylation. We proved the involvement of ZNF536 in neuroendocrine regulation, and identified highly altered ZNF536 as a potential biomarker for immunotherapy.

**Supplementary Information:**

The online version contains supplementary material available at 10.1186/s40001-024-01792-w.

## Introduction

Accurate and precise biomarkers play a crucial role in the field of clinical precision medicine. The Cancer Genome Atlas (TCGA) consortium has been instrumental in identifying cancer driver factors through multi-omics approaches, while the recent Human Tumor Atlas Network (HTAN) initiative has placed emphasis on achieving subcellular resolution. As a whole, data-driven bioinformatics has greatly enhanced the study of target molecules and their interactions, leading to a better understanding of cancer initiation and progression [1].

The diagnosis of neuroendocrine neoplasms relies on the morphological features of the tumor, which are further confirmed through immunohistochemical staining for neuroendocrine markers. Neuroendocrine plasticity is a common characteristic across various anatomical organs. Among them, small cell lung cancer (SCLC) is the most extensively studied poor-differentiated neuroendocrine tumor. In addition, neuroendocrine transformation can occur in lung adenocarcinoma (LUAD) and prostate cancer refractory to targeted therapies [2]. Actually, there is an unmet need regarding molecular driver factors of neuroendocrine transformation.

ZNF536, a gene located on chromosome 19q12, mediated neuronal differentiation and contains multiple C2H2 zinc fingers [3]. The RNA expression levels of ZNF536 may have functional implications. We noted elevated levels of ZNF536 in neuroendocrine prostate cancer, suggesting a potential similarity in the mechanism between LUAD and SCLC [4]. Except for lineage-specific RNA, there are other forms of alterations. A recent study has demonstrated that ZNF536 amplification is associated with poor prognosis in high-grade serous ovarian cancer [5]. Combined with our previous work, mutant ZNF536 associated with total tumor mutational load (TMB) are promising markers for immune checkpoint inhibitor (ICI) therapies in LUAD [6]. However, the precise function of ZNF536, particularly in the context of cancer, remains largely uncharacterized.

In our research, we have undertaken a comprehensive analysis of ZNF536 functionality using a multi-omics approach. We have examined its expression, methylation, mutations, and copy number alterations. Additionally, through high-dimensional single-cell analysis, we have investigated its localization, which has been experimentally validated.

## Methods

### Data acquisition and processing

The multi-omics datasets including RNA expression, methylation, and copy number alterations for TCGA pan-cancer were acquired from Xena website (http://xena.ucsc.edu/), which described the details of processing the data [7]. Specifically, the RNA-seq data were quantified using Transcripts Per Million. As for DNA methylation, the beta value was the ratio of the ratio of methylated to unmethylated densities, while copy number analysis utilized focal scores from genes. Furthermore, mutational and prognostic information of ZNF536 in all cohorts were downloaded from cBioportal portal (https://www.cbioportal.org/) except to Stand Up To Cancer-Mark Foundation (SU2C-MARK) [8, 9]. In details, we investigated the cBioportal searching using subject headings including ‘pan-cancer’ and ‘immunogenomic’. To study the potential regulation of ZNF536, cell lines for small cell lung cancer were used including RNA expression and methylation data [10, 11]. We used 36 common methylated probes from ZNF536 to identify potential target genes through correlation analysis.

For ZNF536 copy number alterations, positive and negative 2 are highly amplified and deleted, respectively [7]. Meanwhile, non-synonymous types including missense, nonsense, and indels were considered mutant. In pan-cancer analysis, we included primary tumor cohorts with over 5 alterations and excluded samples exhibiting concurrent mutations or amplifications. Mutations in TCGA-ovarian cancer (OV) contained frequent amplifications and were therefore removed. Due to the need for lung cancer research, for example, SU2C-MARK, this study did not consider ZNF536-deleted samples. Additionally, mutations and copy number alterations were processed using the MC3 project calling (https://gdc.cancer.gov/about-data/publications/mc3-2017) and GISTIC2 software, respectively. Note that cohorts with prognostic significance are described in Additional file 1: Table S1.

### Methylation analysis of ZNF536 CpG loci

Beta values were used for evaluate methylation levels. To compare the methylation levels of ZNF536 between normal and tumor samples, we used the SMART tool (http://www.bioinfo-zs.com/smartapp) [12]. The DNMIVD website (http://www.unimd.org/dnmivd/) supported the generation of diagnostic and prognostic models, 12 probes were obtained based on the moderate positive correlation with ZNF536 [13]. Furthermore, by calculating the importance scores using XGBoost, cg14914220 was excluded. Integrated probes may be more robust than individual ones, and their mean expression levels and prognosis were analyzed.

### Mutational and copy number alterations related to ZNF536

The **maftools** R package was used to visualize the genomic profile in the SU2C-MARK lung cancer cohort [14]. The two functions including mutant interactions and oncogenic pathways, grouped according to the ZNF536 mutant and wild types. The OncoPrint function was conducted to present copy number alterations genes of 19q12 focal-level in this study. Amplification, deletion and no alterations, were arranged and assigned distinct colors. In addition, the mutational sites of ZNF536 and its structural domains in the International Cancer Genome Consortium (ICGC) data were labeled [15]. Meanwhile, the proportion of the COSMIC website (https://cancer.sanger.ac.uk/cosmic/), which stored the pan-cancer genome, was used to document the types of mutations in ZNF536 [16].

### Immune cell characteristics by deconvolution

For immune cell infiltration, gene expression was as input data imported into ImmuneCellAI website (http://bioinfo.life.hust.edu.cn/ImmuCellAI#!/) [17]. ImmuneCellAI is an online analysis of the relative portion of twenty-four immune cells in particular T cells, and is derived from transcriptome deconvolution. We used ZNF536 alteration as a grouping variable and retained immune cells with p-values less than 0.1. Excluded because there are no meaningful subportions in the TCGA-colon adenocarcinoma (COAD) cohort.

### Pathway (Msigdb and GO) enrichment

**GSVA** R package was used for calculating MsigDB HALLMARK gene set enrichment of each sample by non-parametric analysis (method = “gsva”, mx.diff = FALSE, min.sz = 10) [18, 19]. And, Gene Ontology enrichments were performed using the **clusterProfiler** R package [20]. Three libraries including biological process, cellular component and molecular function were used to annotate genes, which were suggestive of the relevance of ZNF536 methylated loci.

### Single-cell RNA analysis

The HTAN-SCLC processed dataset was downloaded and focused on the cancer epithelium [21]. SCLC epithelium is hypothesized to be regulated by three master transcription factors, ASCL1, NEUROD1 and POU2F3. Particularly, simulated grouping by differential genes between subtypes. The median value of ZNF536 was approximately 0, making it as a rough subgroup marker. Then, methods for identifying co-expressed or co-excluded genes have been detailed in our prior publication [22]. Beyond, we conduct processed analysis by **Seurat** R package [23]. Top 2000 variable genes were chosen for principal component analysis to normalized data using the LogNormalize method. The visualization was then based on the generated reduction using the t-distributed Stochastic Neighbor Embedding.

### Cell culture and transfection

H23, H1944, PC9, and A549 cells were obtained from the Institute of Oncology, Xinqiao Hospital, and all cells were cultured in medium with the addition of 10% fetal bovine serum (FBS; Gibco, Thermo Fisher Scientific) and 1% penicillin/streptomycin solution (37 °C, 5% CO_2_).

For transfection of A549 cells, empty vector and ZNF536 plasmids were constructed and purchased from Shanghai Genechem Co., Ltd. And the original plasmid schematics can be found in the Additional file 2: Figure S1. 1.5 × 10^5^ A549 cells were then seeded in a 6-well plate, and transfections were conducted when the cell confluence reached 70%–90%. Transfection reagents that namely Lipofectamine 3000 (Invitrogen, USA) was purchased from Thermo Fisher Scientific. Transfection took place in culture medium, and the final concentration of plasmid was 5 ng/μl. After transfection for 24–48 h and fluorescence was observed under a fluorescence microscope, the medium was replaced with complete medium containing 2 μg/ml puromycin, and the RNA of the cells was extracted after 24 h.

### Reverse transcription-quantitative PCR (RT-qPCR)

Total RNA was isolated from all cells using RNAiso reagent (Takara, Dalian, China). Reverse transcription was performed according to the instructions of the ExScript TM RT kit (Takara, Dalian, China). Q-PCR reactions were carried out on an ABI 7500 fast real-time PCR system (Foster City, CA, USA), and fold change was calculated using the 2-ΔΔCt method. Cycling conditions were as described previously: 95 °C for 30 s, 95 °C for 5 s, and extension (60 °C, 34 s) for 40 cycles. The expression level of all genes was detected by SYBR Premix Ex Taq II (Takara Biotechnology), and mRNA expression levels were calculated and normalized to GAPDH expression levels. Primer sequences were customised according to NCBI genebank, then acquired from Beijing Tsingke Biotech Co and detailed in Additional file 1: Table S2.

### Statistical analysis

Three independent repetitions were performed for all experiments. GraphPad Prism 8.0 (GraphPad Software, San Diego, CA, USA) was applied to analyze the RT-qPCR data and generate graphs. *p* value less than 0.05 was statistically significant. Survival analysis included Kaplan–Meier and ROC, and were plotted through **survminer** and **pROC** R package, respectively. The non-parametric or Fisher’s test was used to compare all variables. Pearson correlation coefficients were performed, and the results were visualized using a heatmap. All scripts utilized for analyses were written using the R programming language.

## Results

### ZNF536 amplification affects its expression, signature and prognosis

**Fig. 1:**
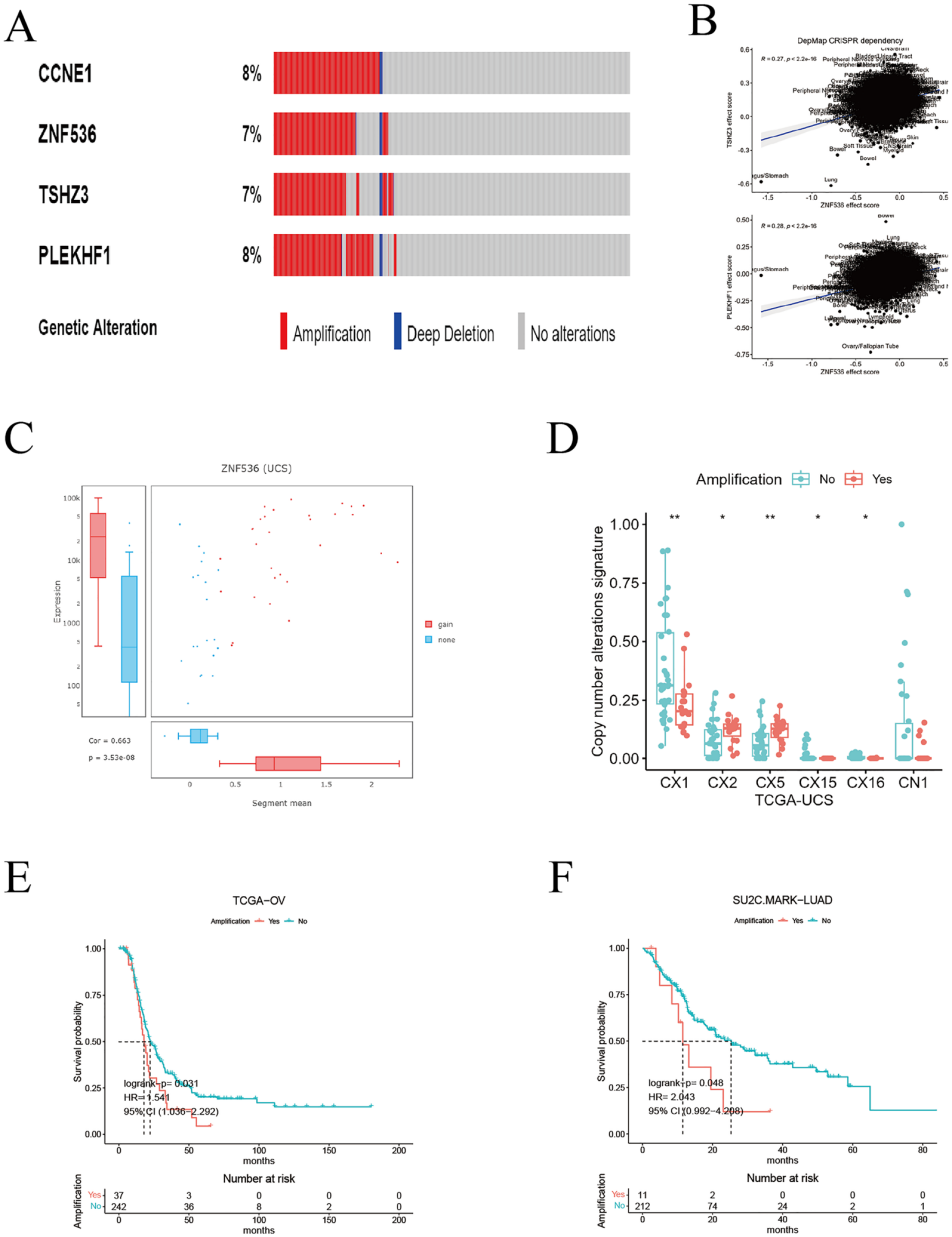
Copy number alteration patterns of ZNF536. **A** Copy number alterations of CCNE1, ZNF536, TSHZ3 and PLEKHF1 using ICGC pan-cancer data on the cBioportal website (https://www.cbioportal.org/). **B** Correlation analyses between ZNF536 and TSHZ3, ZNF536 and PLEKHF1, supported by the DepMap website (https://depmap.org/). **C** Distribution analysis of fragment copy number and RNA expression of ZNF536 using TCGA-UCS data on the DriverDB website (http://driverdb.bioinfomics.org/). **D** Boxplot showing copy number alteration signatures of ZNF536 amplification and wild-type using TCGA-UCS data. Significance levels are denoted by * and ** for *p* < 0.05 and *p* < 0.01, respectively. **E**–**F** Disease-free survival and overall survival grouped by ZNF536 amplification in TCGA-OV and SU2C-MARK_LUAD cohorts

To explore the alterations of ZNF536 across different cancer types, we utilized the cBioportal website for a pan-cancer analysis (Additional file 3: Figure S2A). We found that the copy number alteration of ZNF536 was more concentrated in specific cancers compared to mutation. For example, uterine carcinosarcoma (UCS) showed a higher frequency of ZNF536 copy number alterations. After investigating genes related to 19q12 focal amplification, CCNE1 is a well-known oncogenic driver, and our analysis suggested that ZNF536, along with TSHZ3 and PLEKHF1, may have bypass effects in relation to CCNE1 (Fig. 1A) [24]. Meanwhile, DepMap website provided knockout cell line data to support synthetic lethality [25]. This analysis indicated that ZNF536 was most positively associated with TSHZ3 and PLEKHF1 (Fig. 1B). Overall, the driver of 19q12 amplification can be partly reflected by CRISPR gene editing.

Then, the DriverDB portal identifies the ZNF536 as an amplification driver in the UCS [26]. Furthermore, the fragment values of ZNF536 showed a significant positive correlation with RNA expression (Fig. 1C). Analyzing the aneuploidy diversity, we found that ZNF536 amplification was associated with upregulation of copy number signature CX2 and CX5, while downregulating CX1, CX15, CX16, and CN1 (Fig. 1D) [27]. Although the down-regulation of CN1 did not reach statistical significance, ZNF536 amplification was still associated with distinct chromosome changes. Dysregulation of homologous recombination may be the main mechanism underlying ZNF536 alterations, as suggested by our analysis. Supporting this point, ZNF536 amplification was significant in OV characterized by homologous recombination defects (Fig. 1E) [27]. Additionally, ZNF536 amplification was associated with poor prognosis in LUAD patients treated with ICIs (Fig. 1F).

### ZNF536 mutation grouping differentiates prognosis, co-mutant patterns and oncogenic signaling

**Fig. 2:**
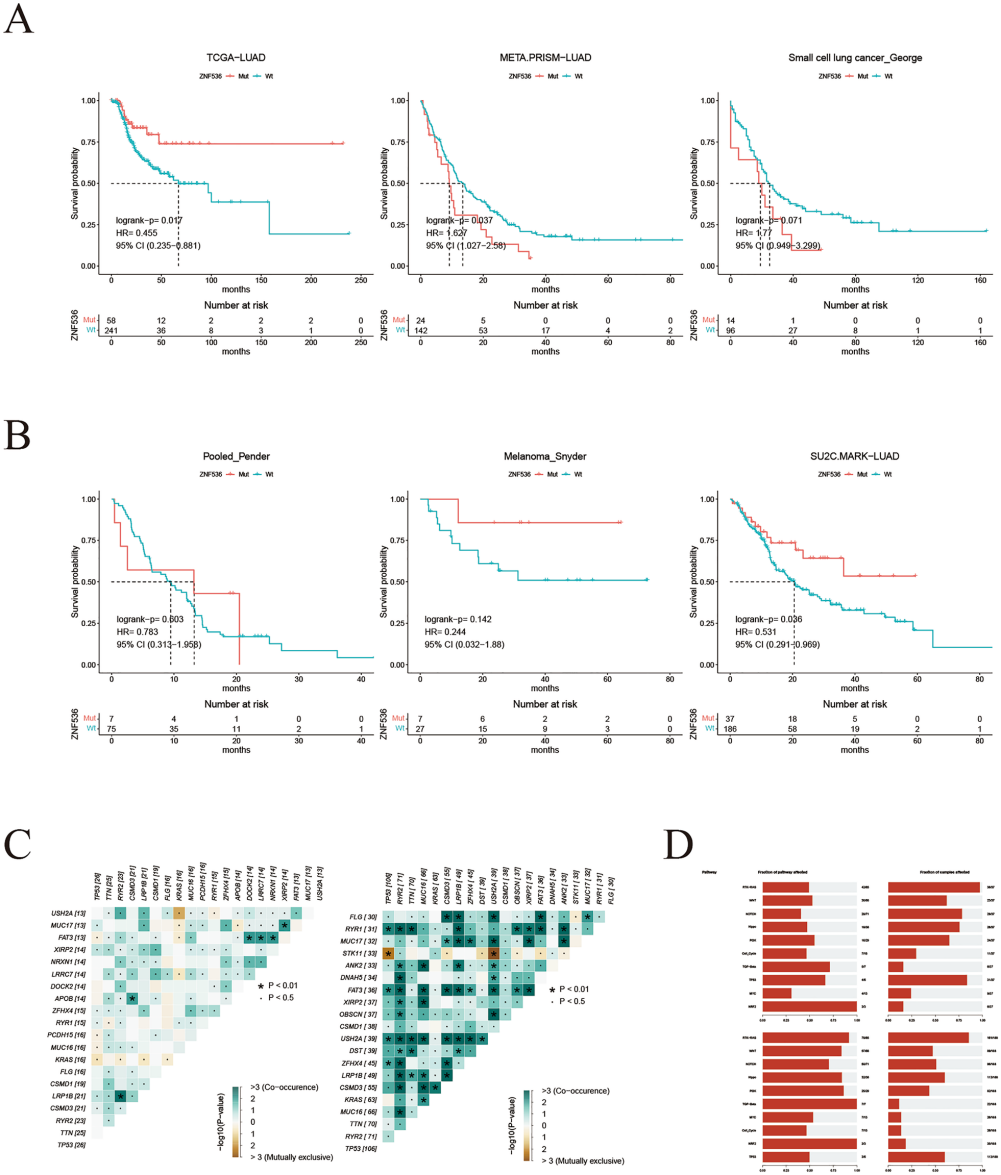
Prognosis and mutational landscape of tumors with ZNF536 mutation. **A** Disease-free survival and overall survival grouped by ZNF536 mutation in lung cancer cohorts, including TCGA-LUAD, META-PRISM_LUAD, and small cell lung cancer_George. **B** Kaplan–Meier plot showing overall survival grouped by ZNF536 mutation in ICI-treated cohorts including Pooled_Pender, Melanoma_Snyder, and SU2C-MARK_LUAD. **C** Waterfall plot displaying the top 20 mutated genes in the SU2C-MARK_LUAD cohort (left: ZNF536-mutant; right: ZNF536-wild). **D** Barplot showing alterations in oncogenic pathways in the SU2C-MARK_LUAD cohort (top: ZNF536-mutant; bottom: ZNF536-wild)

In addition to ZNF536 amplification, we also investigated the mutation in ZNF536. The ICGC pan-cancer profile revealed that ZNF536 mutations and copy number alterations could occur in the same sample, with the highest frequency observed in non-small cell lung cancer (Additional file 3: Figure S2A) [15]. The mutation in ZNF536 were distributed across various sites, and silent type accounted for 18.13% portion, as observed in the COSMIC website (Additional file 3: Figure S2B, C) [16]. When examining the prognostic significance of ZNF536 mutation, we found that their impact varied in primary and metastatic LUAD [1, 28]. Additionally, both metastatic LUAD and SCLC showed poor prognostic value associated with ZNF536 mutation (Fig. 2A) [28, 29]. Similar to the previous study [30], ZNF536 mutation exhibited favorable prognostic value in the Canadian pan-cancer cohort primarily treated with PD-1 blockade [31]. And it was also identified as a favorable prognostic marker in melanoma patients receiving CTLA-4 therapy (Fig. 2B) [32]. This favorable prognostic value may be attributed to the high TMB caused by ZNF536 mutation.

The SU2C-MARK cohort, which is the largest lung cancer cohort receiving ICI therapies, recently provided insights into the transcriptome and genome [9]. In this cohort, ZNF536 showed statistically significant prognostic value in LUAD (Fig. 2B). To further explore ZNF536 mutation, we utilized the **maftools** R package to group co-mutations and analyze oncogenic signaling. Interestingly, we observed mutually exclusive mutations in the genes KRAS and STK11 in the ZNF536-mutant and wild-type groups, respectively (Fig. 2C). Interestingly, the mutations in the wild-type group of ZNF536 were more statistically significant. Furthermore, ZNF536-mutant samples exhibited a higher percentage of pathway alterations compared to the ZNF536 wild-type group, but the pathways affected in the ZNF536 wild-type group were more radically altered, e.g., RTK-RAS (Fig. 2D). In summary, we describe the genomic landscape of ZNF536 mutation and show its prognostic value.

### Pathway and immune cell profiles from ZNF536 alterations

**Fig. 3:**
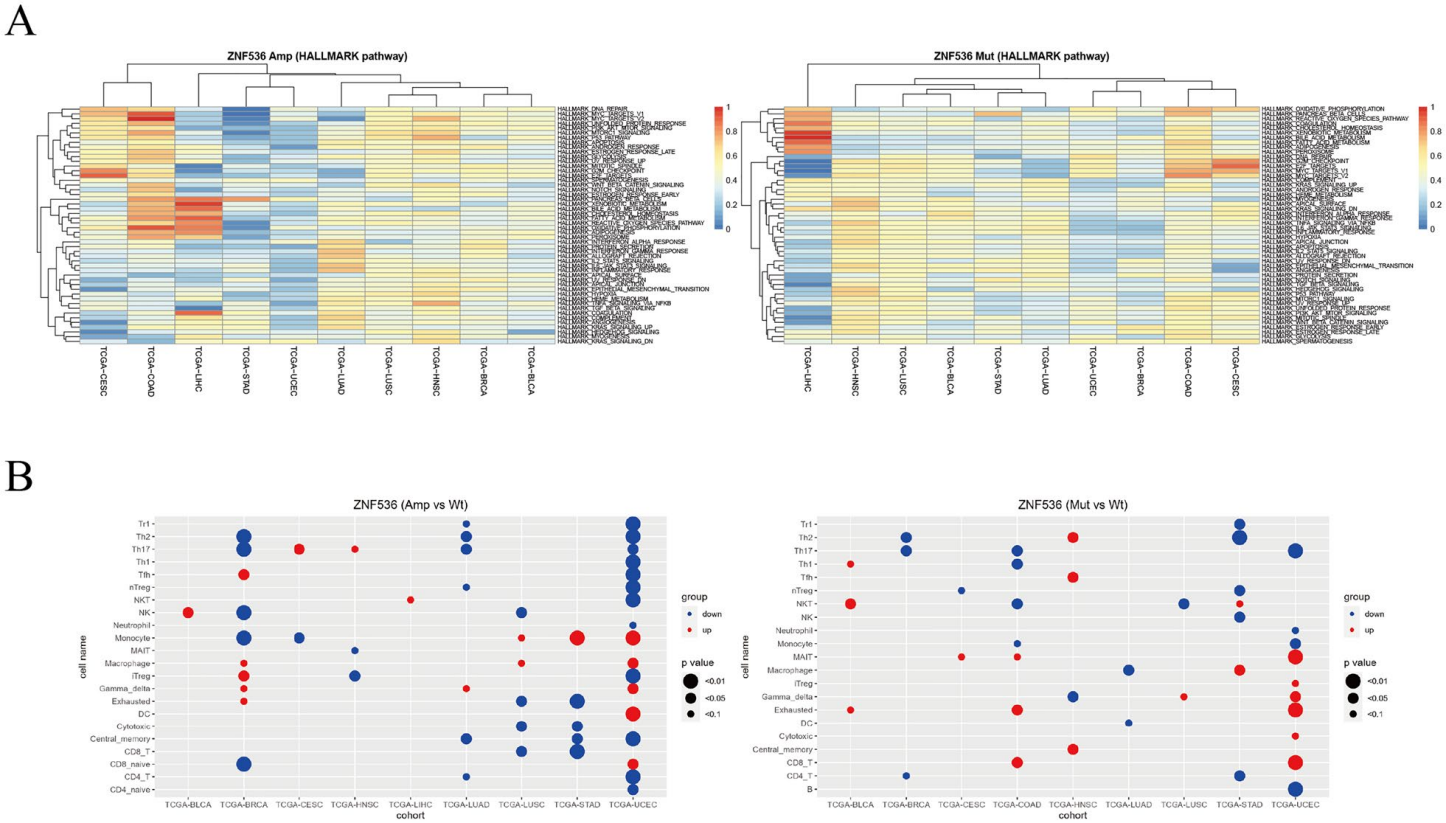
ZNF536 alterations shaping pathways and tumor microenvironment. **A** Enrichment of HALLMARK pathways between ZNF536 amplification and ZNF536 mutation in TCGA pan-cancer data across ten different types. Pathway activity ranges from (0,1), and high values are active. **B** Regulation of immune cell proportions by ZNF536 amplification and ZNF536 mutation in TCGA pan-cancer data across nine different types

In addition to the above analysis, we further evaluated the hallmark pathway in ZNF536-altered cancers based on their RNA expression profiles [18, 19]. In COAD, ZNF536 amplification showed a more activated pathway compared to mutant species. Interestingly, despite the differences in ZNF536 mutation and copy number alterations, COAD and cervical squamous cell carcinoma exhibited similar pathway activities. On the other hand, liver hepatocellular carcinoma appeared to have distinct pathway enrichment, mainly related to metabolic and developmental disorders such as bile acid metabolism, fatty acid metabolism, xenobiotic metabolism, cholesterol homeostasis, oxidative phosphorylation, adipogenesis, and pancreas beta-cell function (Fig. 3A).

We also predicted the immune microenvironment using ImmuCellAI for the aforementioned nine tumor types, excluding COAD [17]. Our analysis highlighted three types of tumor subgroups: central memory and Th2 T-cell subgroups were downregulated by ZNF536 amplification. Notably, we found that ZNF536 mutation upregulated exhausted T cells, which are considered key components in immunotherapy. Meanwhile, MAIT and Th17 T-subsets were upregulated and downregulated by the ZNF536 mutation, respectively (Fig. 3B). These findings suggest that ZNF536 alterations can have different effects on the biological functions of these tumors.

### RNA analysis reveals ZNF536 sublocalization and candidate regulator

We examined the RNA expression of ZNF536 using the GEPIA2 and GTEx portals. GEPIA2 is a website that stores TCGA data, while GTEx is non-redundant and does not incorporate cancer data [33, 34]. GEPIA2 exhibited that ZNF536 was predominantly expressed in glioma and pheochromocytoma and paraganglioma, and the two main isoforms (ENST00000355537.3 and ENST00000592773.2) showed a similar expression pattern (Additional file 4: Figure S3A). Additionally, ZNF536 was found to be expressed in most normal tissues, with a higher expression level in the brain according to the GTEx portal (Additional file 4: Figure S3B).

**Fig. 4:**
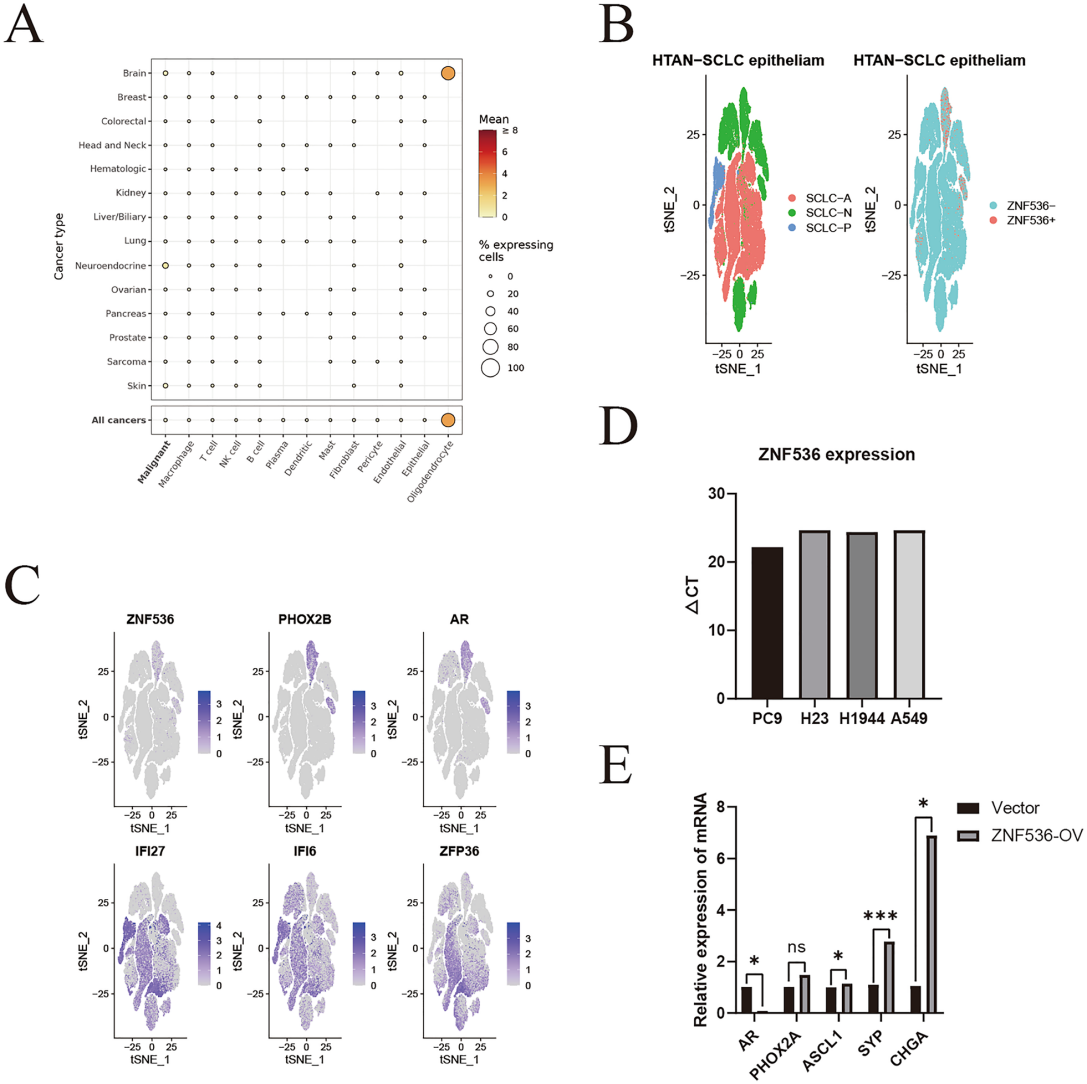
Potential upstream and downstream regulators of ZNF536. **A**, **B** Analysis of single-cell RNA expression of ZNF536 in Curated Cancer Cell Atlas and HTAN-SCLC datasets. **C** *t*-distributed Stochastic Neighbor Embedding (tSNE) plot illustrating the relative distribution of ZNF536, PHOX2B, AR, IFI27, IFI6, and ZFP36 in the HTAN-SCLC dataset (top: co-expression; bottom: co-excluded). **D** RT-qPCR assay of ZNF536 expression in PC9, NCI-H23, NCI-H1944 and A549 lung cancer cell lines. **E** Transcription factors and neuroendocrine markers regulated by ZNF536 (vector: empty; ZNF536-OV: ZNF536-overexpressed, repetitions: three times). Significance levels are denoted by * and *** for *p* < 0.05 and *p* < 0.001, respectively

To gain further insights, we investigated high-resolution single-cell RNA pan-cancer profiles [35]. Our observations revealed that ZNF536 was predominantly expressed in oligodendroglial cells of the brain, which aligns with previous descriptions (Fig. 4A) [36]. Furthermore, analysis of the HTAN-SCLC dataset showed that ZNF536 was primarily expressed in the cancer epithelium and not in the ASCL1^+^ subpopulation (Fig. 4B). When grouped by ZNF536 expression, we found that in the cancer epithelium, ZNF536 exhibited co-expression with PHOX2B and AR, while being excluded from IFI27, IFI6, and ZFP36 (Fig. 4C).

Subsequently, we validated the expression and targets of ZNF536 using RT-qPCR. The RNA expression of ZNF536 was extremely low in lung cancer cell lines, so we constructed one of these lines with overexpressed ZNF536 (Fig. 4D). Our results showed that ZNF536 downregulated androgen receptor (AR) but upregulated typical neuroendocrine markers (CHGA and SYP). Compared to this, there was only a slight increase in transcription factors (ASCL1 and PHOX2A, Fig. 4E).

### Correlation, expression, involved pathways, diagnosis and prognosis of methylated ZNF536

**Fig. 5:**
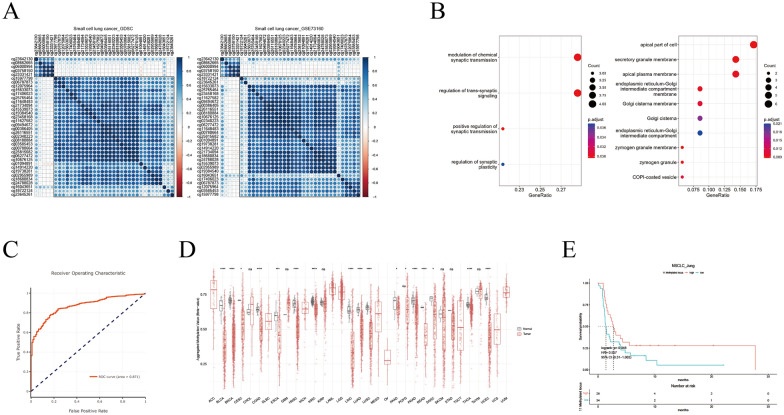
Comprehensive analysis of ZNF536-associated CpG probes with focus on their integration level. **A** Correlation heatmap showing two clusters of ZNF536-related CpG loci in SCLC cell lines (GDSC and GSE73160). **B** Gene Ontology item enrichment using genes associated with ZNF536-related CpG loci (left: positively correlation; right: negatively correlation). **C**, **D** Receiver operating characteristic (ROC) diagnostic curve and distribution of aggregated CpG loci using TCGA pan-cancer data on the DNMIVD website (http://www.unimd.org/dnmivd/). **E** Kaplan–Meier plot of aggregated CpG loci in the GSE119144 cohort based on appropriate cut points

Finally, SCLC lung cancer cell lines were used to study the potential regulation of ZNF536 [10, 11]. In comparing datasets, we found that certain probes (cg08662665, cg06000994, cg03758150, and cg23331421) in the 5'UTR region of ZNF536 were inconsistent with the rest of the probes (*r*_mean_ < 0.3, Fig. 5A). We assumed that the remaining 31 probes co-associated genes as regulated genes. Pathway analysis revealed that the positively correlated target genes were involved in synaptic transmission, while the negatively correlated genes were associated with organelles (Fig. 5B). The highly relevant ZNF536 probe was selected and screened by dnmivd website. Integration levels of 11 probes could distinguish between tumor and normal sample (ROC = 0.871, Fig. 5C). We believed that 11 probes reflect a consistent expression pattern of ZNF536, but the remaining 4 probes might be opposite (Fig. 5D, Additional file 5: Figure S4). Importantly, high levels of methylation through appropriate cut points presented a favorable prognostic trend in the GSE119144 cohort (Fig. 5E) [37]. This suggested that increased methylation of ZNF536 might have a positive impact on ICIs efficiency.

## Discussion

This is the first study to characterize the role of ZNF536 in cancer, and our findings regarding its prognostic value, multi-omics analysis, cellular mapping, and immune infiltration regulation are insightful.

Using public datasets, we observed that ZNF536 amplification promotes RNA expression UCS. Consistent with recent findings, ZNF536 amplification was associated with poor prognosis in OV. However, the prognostic significance of ZNF536 mutation appeared to vary across different datasets, potentially due to variations in TMB [6, 30]. By investigating nine tumors with both ZNF536 mutation and copy number alterations, we have identified distinct patterns of immune infiltration regulation. Pathway enrichment analysis suggested that ZNF536 mutation and amplification share similarities but can form distinct clusters. Furthermore, we have elucidated the potential regulatory mechanisms of ZNF536 through methylation and single-cell RNA analyses. The study also explored the role of ZNF536 alterations in immunotherapy, highlighting the various roles played by ZNF536 copy number, mutation, and methylation status. This may be explained by the differential shaping by ZNF536 alterations (Fig. 3A, B).

ZNF536 is located at 19q12 and the drivers of 19q12 amplification are complex [38]. Interestingly, CCNE1 does not affect prognosis whereas ZNF536 did [5]. In ICGC pan-cancer data, ZNF536 alterations were identified in 9.8% of cases. Particularly, about 40% of UCS patients contained high amplification of ZNF536 but without any highly deletions. In SCLC, CCNE1 amplification is frequent and its prognostic significance has been recently demonstrated [39]. Considering the prognostic significance of ZNF536 alterations in SCLC, the inclusion of ZNF536 in in situ hybridization for the 19q12 amplification would be practical. Furthermore, in mutation analysis, the impacts of amplification and silent type need to be considered. In addition, its deletion is rarer than amplification in the TCGA cohort, and the wide peak of 19q12 amplification could contain ZNF536.

SCLC is a type of classical neuroendocrine cancer. There is still a lack of effective therapies to address this highly aggressive cancer. The data-driven bioinformatics helps identify the driver gene to guide cancer therapies. We have shown that alterations in ZNF536 have prognostic significance, such as mutation. ZNF536 is difficult to target directly as a transcription factor, unlike kinases (e.g., FGFR3). Antibody-drug conjugates may be inoperable because ZNF536 can be expressed in normal tissues []. Compared with this, synthetic engineering may be advantageous if gene acts as a master regulator with prognostic significance. In psychiatric disorders, ZNF537/TSHZ3 and ZNF536 were expressed in the cerebral cortex, and supported by CRISPR information [40]. Indeed, targeted design for ZNF536 are beyond the manuscript’s scope.

Then, potential upstream and downstream targets of ZNF536 were explored. Literature analysis does not support ZNF536 as a target of ASCL1 and NEUROD1, suggesting the need for further studies on the crosstalk of transcription factors [41]. Recently, single-cell RNA analysis provided valuable information on sublocalization and co-expression to aid in the identification of regulators. Guided by the HTAN-SCLC cancer epithelium dataset, we further found that the RNA expression of ASCL1, PHOX2A and AR was regulated by ZNF536 (Fig. 4E). Further investigation is required to determine the inverse effect of the aforementioned molecules on ZNF536, i.e., whether they act upstream of ZNF536. Interestingly, ZNF536 downregulated AR but co-expressed with AR. Targeting cancer epithelium is feasible, and we previously evaluated the anti-proliferation effect of enzalutamide in lung squamous carcinoma cell lines, but still have not investigated ZNF536 regulation of AR signaling pathway [42]. Finally, the expression of ZNF536 may be determined by its own copy number, methylation and its nearby non-coding RNAs, thus the regulatory mechanism is likely complex [43].

Positive neuroendocrine markers have been seen in adenocarcinomas, melanomas, etc., but hardly affect prognosis [44]. New molecular profiles, and multidisciplinary approach can help clarify diagnosis and prognosis. Methylated ZNF536 were positively correlated with synaptic pathway related to neuroendocrine tumors. Previously, zinc fingers methylation could be used to diagnose cancer (e.g., ZNF154), we further indicate a potential contribution of methylation to alternative RNA expression [45]. Part zinc finger RNAs in cancer exhibit lineage-specific patterns, while DNA methylation is more readily quantified dynamically and provides the genome information, with ZNF536 serving as an example. Our unpublished research also underscores the impact of DNA methylation alterations on the zinc finger family in cancer, and suggests some websites for co-methylation explorations.

We aimed to characterize the cancer driver genes, and found previous studies of ZNF536 to be misleading [46, 47]. First, the ZNF536 antibody may lack specificity and therefore needs to be combined with mass spectrometry, which may lead to biased results. Similarly, pathway analysis of the ZNF536 mutation confounded patients with high TMB. In contrast, we uncovers the cancer types preferentially validated by ZNF536 (e.g., OV, LUAD and SCLC), and explored potential targets. To our understanding, the advantage of ZNF536 over other zinc finger proteins lies in the diverse altered categories that can be synthesized via agonism or antagonism.

In conclusion, this study provides an integrative analysis of the role of ZNF536 in cancer, with a specific focus on lung cancer. We shed light on its prognostic significance, and potential regulatory mechanisms. Importantly, the targeted sequencing T200 platform had previously incorporated the gene. While the reliance on public datasets for analysis may have limitations, as datasets are often pre-processed with ethical constraints. For instance, synonymous/silent mutational information may not be accessible. The same dataset differs between portals, e.g., IMPACT website is inconsistent with cBioportal. Furthermore, the pan-cancer protein profile of ZNF536 remains to be characterized.

## Conclusion

This research depicted the mutation, copy number alteration, DNA methylation, and RNA expression of ZNF536 based on public datasets. Bioinformatics analysis, substantiated by experimental data, elucidated the role of ZNF536 in neuroendocrine regulation. In addition, alterations in ZNF536 have both diagnostic and prognostic implications.

### Supplementary Information

**Additional file 1: Table S1.** Related cohorts in this study. **Table S2.** qPCR Primers in this study.

**Additional file 2: Figure S1.** Plasmid map for overexpression of ZNF536.

**Additional file 3: Figure S2.** Genetic alterations of ZNF536 in ICGC and COSMIC. B) Genetic alterations and variant amino position of ZNF536 in pan-cancer data from ICGC on cBioportal website (https://www.cbioportal.org/). (C) Pie chart displaying the broadest cancer mutational categories of ZNF536 from COSMIC website (http://cancer.sanger.ac.uk).

**Additional file 4: Figure S3.** RNA expression of ZNF536 in TCGA and GTEx. (A) Isoform expression of ZNF536 in pan-cancer data from TCGA on GEPIA2 website (http://gepia2.cancer-pku.cn/). (B) Bulk RNA expression of ZNF536 in normal samples from the GTEx project (https://www.gtexportal.org/home/).

**Additional file 5: Figure S4.** 5'UTR region of ZNF536 methylation in TCGA. Boxplot showing β values of cg08662665, cg06000994, cg03758150, and cg23331421 in pan-cancer data from TCGA on the SMART website (http://www.bioinfo-zs.com/smartapp). Significance levels are denoted by *, **, ***, and **** for p < 0.05, p < 0.01, p < 0.001, and p < 0.0001, respectively.

## Data Availability

All data generated and methods described were in accordance with the relevant guidelines and are permitted by non-commercial organization and did not need access approval. The corresponding author can be contacted for reasonable data.

## Abbreviations

TCGA: The Cancer Genome Atlas
HTAN: Human Tumor Atlas Network
SCLC: Small cell lung cancer
LUAD: Lung adenocarcinomas
TMB: Tumor mutational load
ICI: Immune checkpoint inhibitor
SU2C-MARK: Stand Up To Cancer-Mark Foundation
OV: Ovarian cancer
ICGC: International Cancer Genome Consortium
COAD: Colon adenocarcinoma
UCS: Uterine carcinosarcoma
AR: Androgen receptor
