# Supplementary figures and images for: Characterization of zinc finger protein 536, a neuroendocrine regulator, using pan-cancer analysis
Source: Eur J Med Res. 2024 May 8;29:273. doi: 10.1186/s40001-024-01792-w (PMC11077744; doi:10.1186/s40001-024-01792-w)

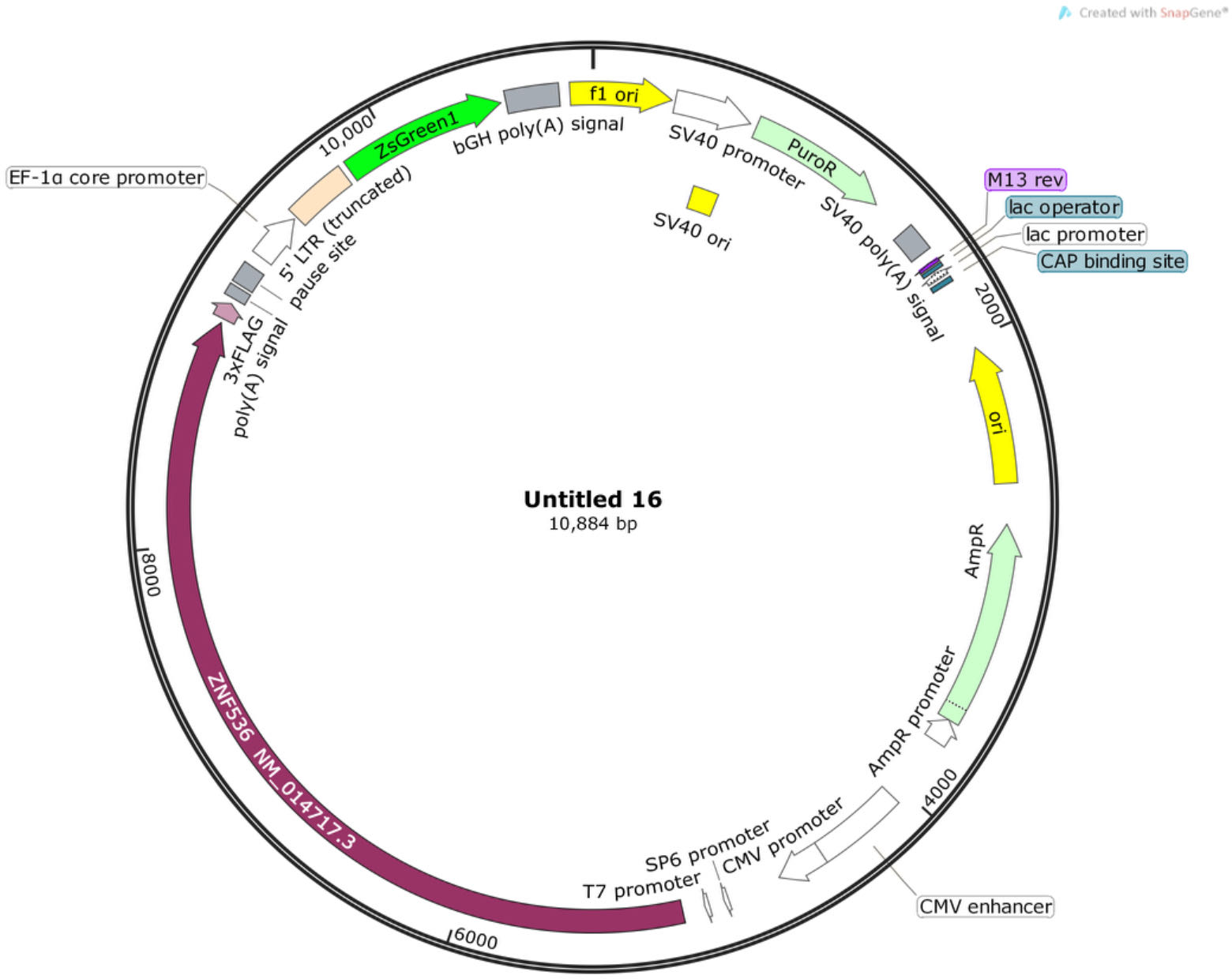

Supplement: Supplementary file 2 — Additional file 2: Figure S1. Plasmid map for overexpression of ZNF536. [file 40001_2024_1792_MOESM2_ESM.tif]

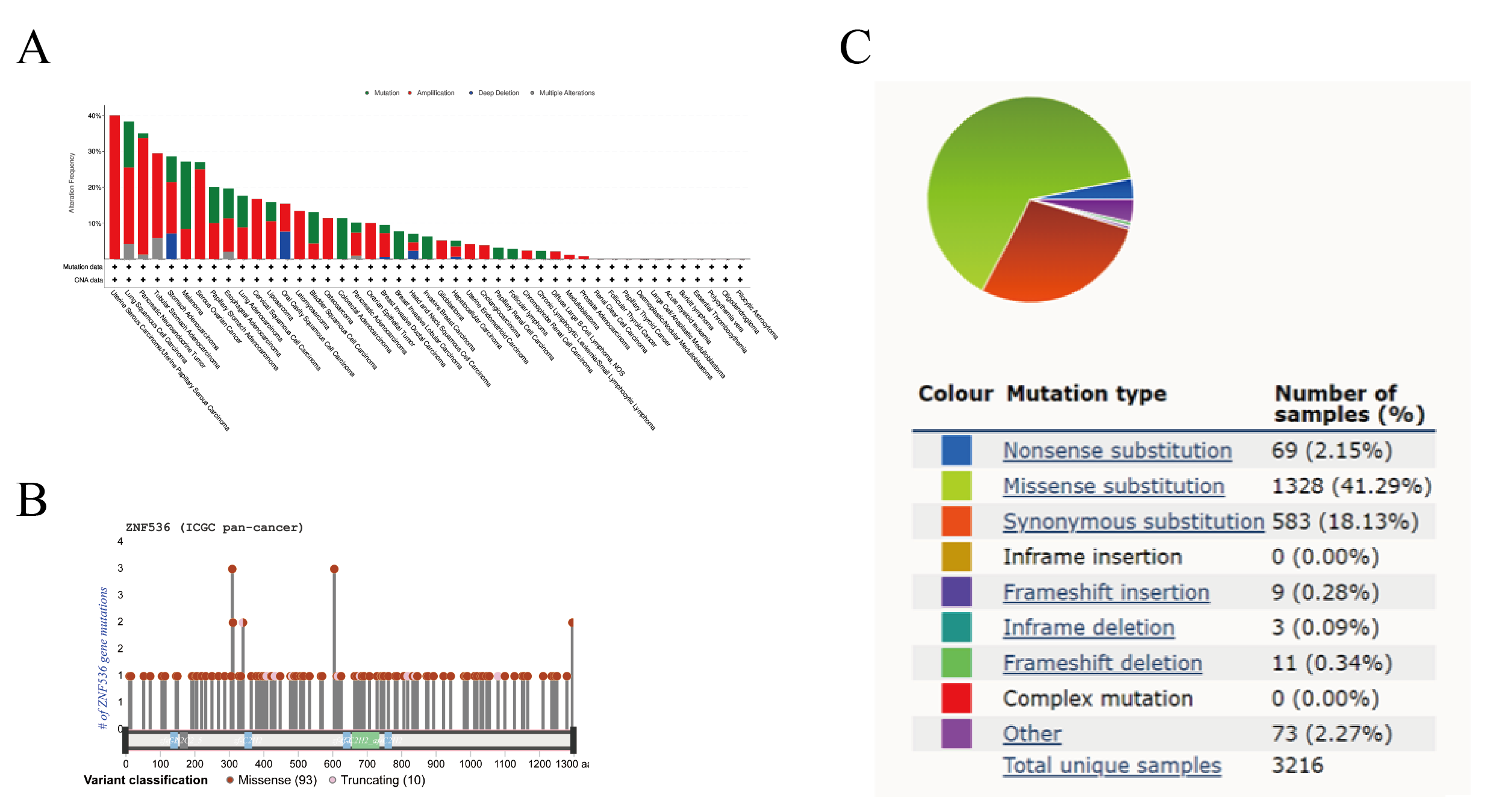

Supplement: Supplementary file 3 — Additional file 3: Figure S2. Genetic alterations of ZNF536 in ICGC and COSMIC. B) Genetic alterations and variant amino position of ZNF536 in pan-cancer data from ICGC on cBioportal website (https://www.cbioportal.org/). (C) Pie chart displaying the broadest cancer mutational categories of ZNF536 from COSMIC website (http://cancer.sanger.ac.uk). [file 40001_2024_1792_MOESM3_ESM.tif]

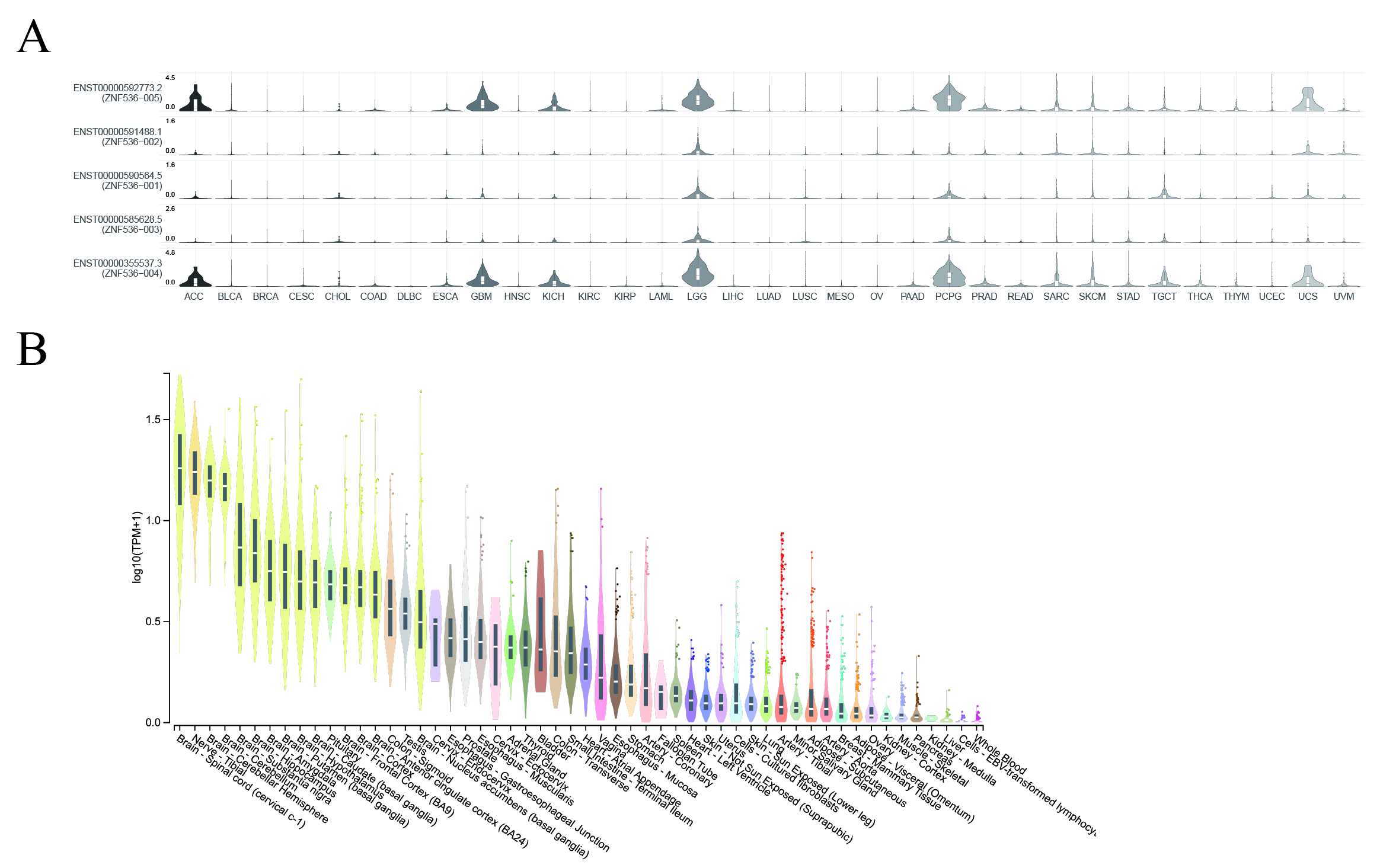

Supplement: Supplementary file 4 — Additional file 4: Figure S3. RNA expression of ZNF536 in TCGA and GTEx. (A) Isoform expression of ZNF536 in pan-cancer data from TCGA on GEPIA2 website (http://gepia2.cancer-pku.cn/). (B) Bulk RNA expression of ZNF536 in normal samples from the GTEx project (https://www.gtexportal.org/home/). [file 40001_2024_1792_MOESM4_ESM.tif]

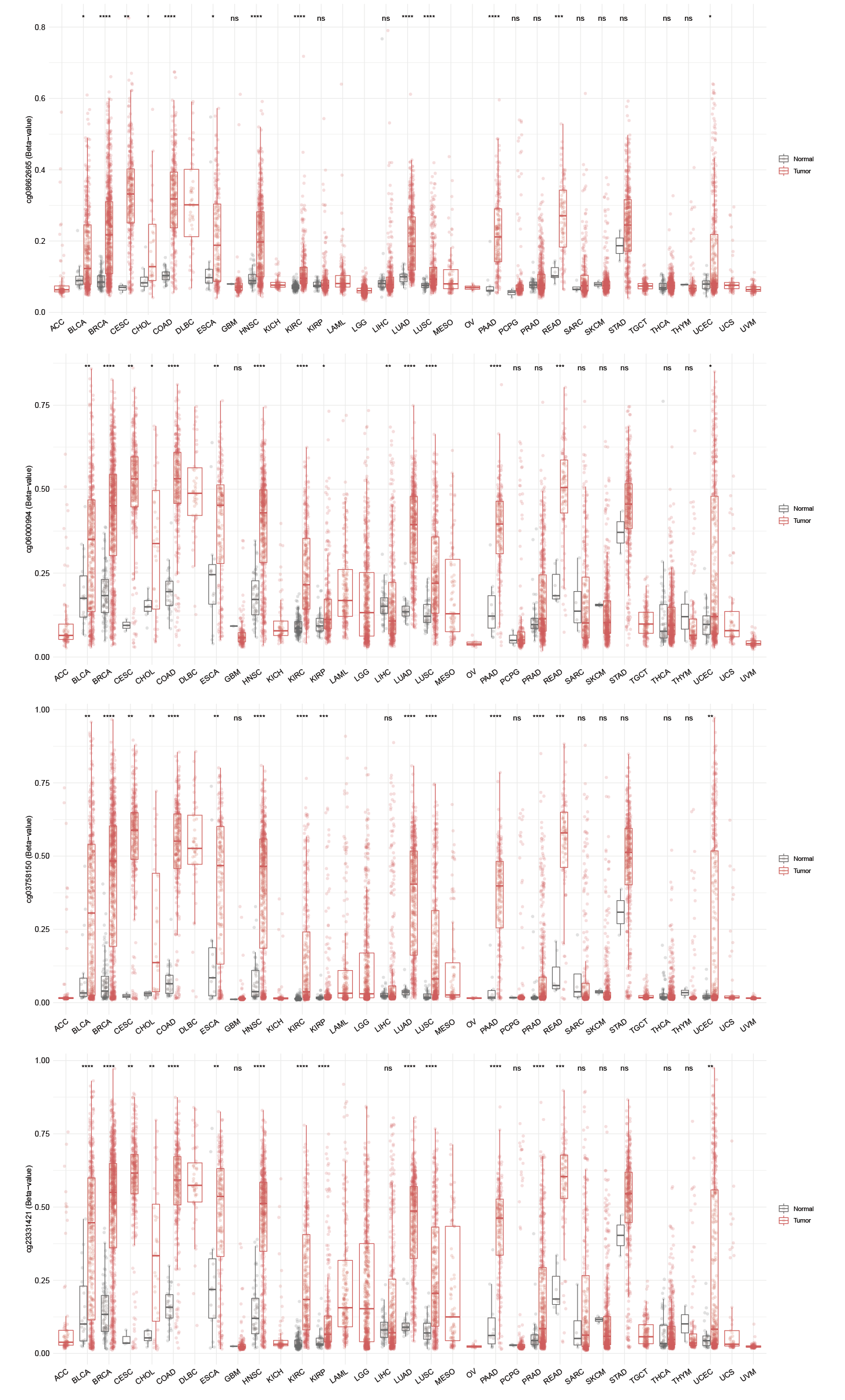

Supplement: Supplementary file 5 — Additional file 5: Figure S4. 5'UTR region of ZNF536 methylation in TCGA. Boxplot showing β values of cg08662665, cg06000994, cg03758150, and cg23331421 in pan-cancer data from TCGA on the SMART website (http://www.bioinfo-zs.com/smartapp). Significance levels are denoted by *, **, ***, and **** for p < 0.05, p < 0.01, p < 0.001, and p < 0.0001, respectively. [file 40001_2024_1792_MOESM5_ESM.tif]
